# Podoconiosis in Rwanda: Knowledge, attitudes and practices among health professionals and environmental officers

**DOI:** 10.1371/journal.pntd.0008740

**Published:** 2020-10-07

**Authors:** Ursin Bayisenge, Janna Schurer, Rex Wong, Hellen Amuguni, Gail Davey

**Affiliations:** 1 Center for One Health, University of Global Health Equity, Kigali, Rwanda; 2 Cummings School of Veterinary Medicine at Tufts University, North Grafton, United States of America; 3 Yale University, Connecticut, United States of America; 4 Centre for Global Health Research, Brighton & Sussex Medical School, Falmer Campus, University of Sussex, Brighton United Kingdom; 5 School of Public Health, Addis Ababa University, Ethiopia; National Institutes of Health, UNITED STATES

## Abstract

**Background:**

Podoconiosis is a neglected tropical disease commonly found in volcanic regions, where soil is rich in silica. It usually manifests as bilateral lower limb edema. The majority of people affected by podoconiosis are farmers who do not wear shoes. The condition was recently documented in all 30 districts in Rwanda but knowledge, attitudes and practices (KAP) of Rwandan health professionals and environmental officers towards podoconiosis are unknown.

**Methodology/Findings:**

The objective of this study was to assess the knowledge, attitudes and practices (KAP) of Rwandan health providers and environmental officers towards podoconiosis in order to improve patient healthcare experiences and health outcomes, and to reduce stigma against affected individuals. To achieve this goal, we administered a KAP assessment to physicians (N = 13), nurses/midwives (N = 59), community health workers (N = 226), and environmental officers (N = 38) in the third highest podoconiosis prevalence district in Rwanda (Musanze).

All 336 respondents had heard of podoconiosis, but 147 (44%) respondents correctly identified soil as the only direct cause of podoconiosis. The awareness of signs and symptoms and risk groups was lower than any other category (31.5% and 47.5%, respectively). The overall attitude toward podoconiosis was positive (86.1%), with CHWs least likely to harbor negative beliefs against podoconiosis patients. One particular area where most respondents (76%) expressed negative attitude was that they saw people with podoconiosis as a threat to their own health and their family’s health. Prescription of antibiotics and use of ointments/soap to manage wounds was low (5% and 32.2%, respectively), in part due to supply shortages at health facilities.

**Conclusions:**

This study identified clear gaps in health provider knowledge and practices that affect patient care for those with podoconiosis. Improved access to essential medicines at health facilities and podoconiosis-focused training sessions for practicing health providers are necessary to minimize the burden and stigma of affected individuals.

## Author summary

Podoconiosis is a foot disease that progressively affects genetically susceptible people who do not wear shoes in volcanic soil. This disease causes disability, stigma, and impinges on people’s economic productivity. In Rwanda, it is estimated that 6429 people live with podoconiosis, but health services for these particular patients are almost nonexistent. We evaluated knowledge, attitudes and practice among 298 health professionals and 38 environmental officers and found that only 44% of all respondents knew that soil is the only direct cause of podoconiosis. Few health professionals (6%) reported having treated a podoconiosis patient and most health professionals (79%) identified shortages of critical drugs and supplies as an important barrier to providing treatment. Three quarters of environmental officers (74%) did not know that farmers are the people most vulnerable to this condition. The poor knowledge of all respondents toward podoconiosis and the lack of medical supplies suggest that podoconiosis patients likely receive substandard medical care and receive little credible information on prevention. We recommend a multi-sectoral approach to training, as well as the engagement of governments and the World Health Organization, to avail medications and treatment materials in the community.

## Introduction

Podoconiosis is a Neglected Tropical Disease (NTD) that occurs in volcanic and tropical regions with heavy precipitation and high altitude where genetically-susceptible people are exposed to irritant soils rich in silica [1,2]. Those at highest risk are low income farmers who do not wear shoes and whose feet are continually exposed to irritant red clay soils [3].

Podoconiosis is characterized by progressive bilateral lower limb edema. Since the condition is caused by long-term soil exposure, it can appear in those as young as 15 years of age but usually does not manifest until the third decade of life [4,5]. Affected individuals experience at least five acute attacks per year, during which patients experience episodes of painful inflammation in affected legs [6]. These attacks prevent patients from walking and result in up to 90 lost work days per year [6]. In addition to physical effects, affected individuals experience stigma such as social exclusion, poor marriage prospects, and poor medical care [5]. This community marginalization results in school dropout, minimal or inexistent engagement in community leadership, among others [5]. Furthermore, podoconiosis causes an economic burden because it affects people during their most productive years.

Although podoconiosis is officially recognized by the World Health Organization (WHO) as an NTD, there is no global roadmap to guide prevention or control. Podoconiosis is still placed under the lymphatic filariasis (LF) control program–which leaves countries like Rwanda with no LF program in a challenging situation [1,7]. Podoconiosis is endemic to 32 countries (18 in Africa, 3 in Asia, and 11 in Latin America) [8], with most cases occurring in sub-Saharan Africa [9]. Podoconiosis is endemic to Rwanda with an estimated prevalence of 68.5 cases per 100,000 people [10]. The highest prevalence occurs in the Northern and Western Provinces [10].

Very little information is available regarding the health experiences of podoconiosis patients in Rwanda; however, stigma and poor health provider knowledge have been identified as important barriers to quality care in nearby Ethiopia [11]. In June 2018, the Rwanda Ministry of Health conducted a 2-day countrywide training for community health workers (CHWs) on NTDs—including podoconiosis—but no knowledge assessment was conducted to evaluate the impact of the training. The objective of this study was to assess the knowledge, attitudes, and practices (KAP) of health professionals and environmental officers towards podoconiosis in Northern Rwanda.

## Methods

### Setting

**Fig 1:**
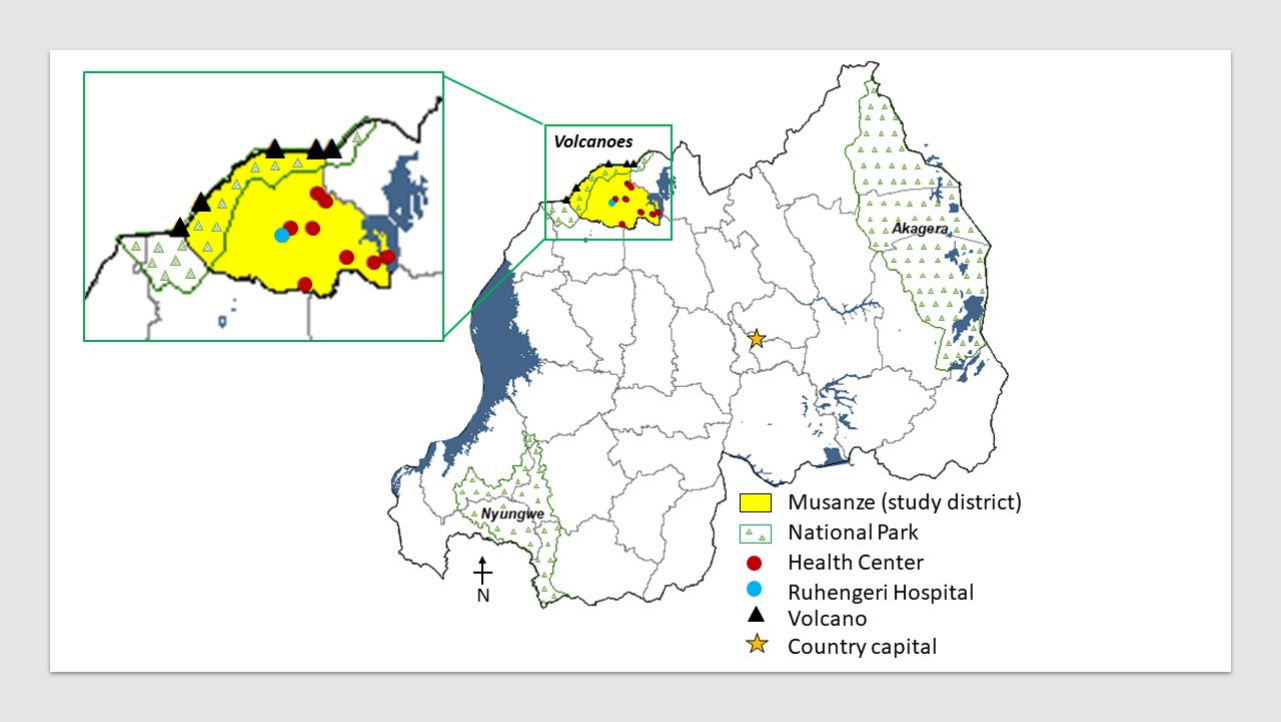
Proximity of podoconiosis study sites (health centers and provincial hospital) to volcanoes in Musanze District, Rwanda, ArcGIS v10.6.1.

Rwanda (also named ‘the land of a thousand hills’) is located in East Africa and is bordered by Uganda, Burundi, the Democratic Republic of the Congo, and Tanzania. Rwanda is subdivided into 4 provinces (North, South, East and West) as well as the city of Kigali. This study was conducted in Musanze District, Northern Province, which has the third highest podoconiosis prevalence in Rwanda (100.9 cases per 100,000 people) (Fig 1) [10]. Currently more than 300 patients are estimated to suffer from podoconiosis in the study district [10]. Musanze has a population of 416,000 residents [12], of which 91% are farmers [13]. The presence of five volcanoes in this district makes the soil of this region rich in silica, which accumulate from the disintegration of larva in regions of high altitude [14]. Precipitation varies between 1,400 mm and 1,800 mm throughout the year with a mean temperature of 20 degrees Celsius [15]. Therefore, environmental conditions and agricultural activities in this region are favorable to podoconiosis occurrence.

**Fig 2:**
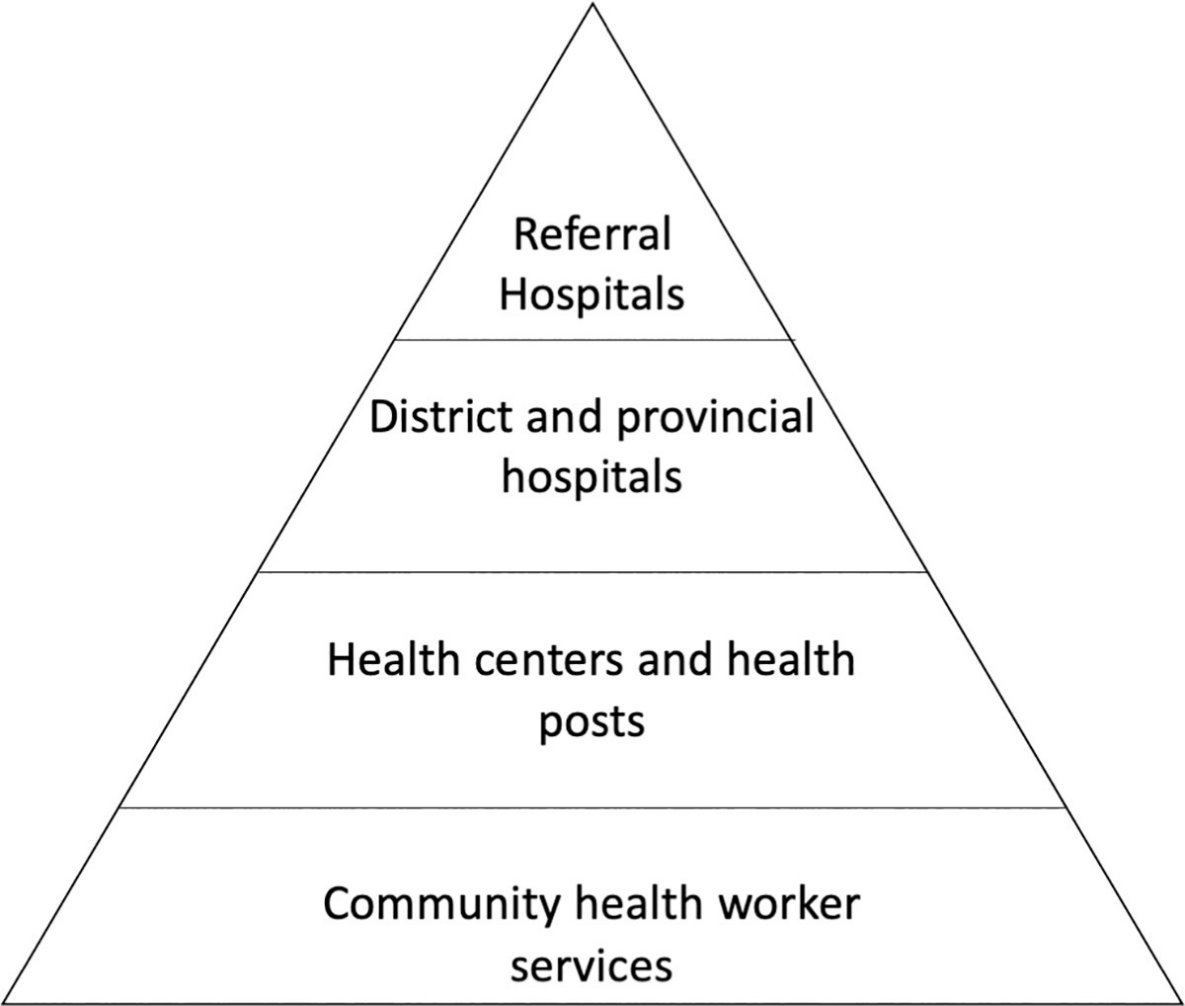
Hierarchy of services provided by the Rwandan health system.

The Rwandan health system follows a hierarchical structure starting with services offered by CHWs at the village level leading up to health posts/centers, district/provincial hospitals, and ending with referral/teaching hospitals at the national level (Fig 2) [16]. Environmental officers work at sector and district government offices, with roles that include Director of Agricultural and Natural Resources, livestock officers, environmental officers, agronomists, veterinary officers and Coordinator of Agricultural Cooperatives.

### Design

In 2018 we conducted a cross-sectional assessment of KAP among physicians, nurses, midwives, CHWs, and environmental officers.

At that time, there were 25 doctors, 284 nurses, 22 midwives, 1728 CHWs, and 49 environmental officers working in Musanze District. All physicians and environmental officers in the study area were invited to participate. We administered the questionnaire to 50% of nurses who worked during the day shift during the study period at Ruhengeri Referral Hospital. To recruit CHWs, we randomly selected eight health centers and requested lists of all CHWs overseen by each center. Thirty CHWs from each of the eight centers were then randomly invited to participate. In total, 374 participants were invited to participate in this study.

### Data collection tool and method

We adapted a questionnaire used to assess the KAP of health professionals toward podoconiosis in Ethiopia [11]. We customized the questionnaire by including choices relevant to CHWs and environmental officers. Color pictures illustrating different clinical stages of podoconiosis were integrated into the survey to minimize misunderstanding of the disease. The questionnaire was pre-tested on three healthcare providers and two environmental officers in Kigali and modified accordingly. The questionnaire was uploaded to Kobo Toolbox and administered on smartphones by trained data collectors in a private office at respondents’ workplaces. The questionnaire took approximately 15 minutes to complete and respondents could choose to answer in English or Kinyarwanda, according to their preference.

### Statistical analysis

Three key measures of this study were: 1) knowledge score per person, 2) percentage of positive and negative attitudes toward podoconiosis, and 3) scores of podoconiosis management practices. All scores were categorized as ‘poor’ or ‘negative’ if below 80% and ‘good’ or ‘positive’ if equal to or above 80%, [17]. Attitude questions were considered negative when they implied a devaluing connotation or stigma for podoconiosis patients, and considered positive when they implied good self-esteem or valuing demeanor for podoconiosis patients.

All data were entered into Excel and cleaned before being exported into SPSS version 17.0 for analysis. Associations between demographic variables and KAP were evaluated using the Fisher Exact test, with p-value set at 0.05.

### Ethics

The study was reviewed and approved by the Institutional Review Board (ID: 0065) of the University of Global Health Equity. Written permission to carry out studies was also sought and received from government leaders in Musanze District and the administrators of Ruhengeri Referral Hospital. Written consent was obtained from all participants.

## Results

### Demographic characteristics of the study participants

Of the 336 respondents who participated in this study, 196 (58%) were female and 140 (42%) were male (Table 1). The participation rate was 90%. Participants’ age ranged from 22 to 70 years old (mean 43 years) with work experience ranging from 1 to 35 years (mean 10 years). Most participants (61%, n = 204) had completed primary education and 30% (n = 103) had completed university level education. Thirteen participants (4%) were physicians, 59 (18%) were nurses and midwives, 226 (67%) were CHWs and 38 (11%) were environmental officers. Among health professionals, only 19 (6%) had seen at least one case of podoconiosis each month at their workplace (Table 1).

**Table 1:** Demographic characteristics of study participants surveyed on knowledge, attitudes and practices of podoconiosis in Musanze District, Rwanda.

Frequency N (%)				
Variable	FHCP^1^	CHWs^2^	Environmental officers	All
Sample (N)	72	226	38	336
**Age (years)**				
< 40	50 (69)	54 (24)	27 (71)	131 (39)
40–59	21 (29)	145 (64)	11(29)	177 (53)
≥ 60	1 (1)	27 (12)	0 (0)	28 (8)
**Sex**				
Male	27 (37.5)	81 (36)	32 (84)	140 (42)
Female	45 (62.5)	145 (64)	6 (16)	196 (58)
**Experience (years)**				
< 10	38 (53)	81 (36)	33 (87)	152 (45)
10 to 19	22 (30)	129 (57)	5 (13)	156 (46)
≥ 20	12 (17)	16 (7)	0 (0)	28 (8)
**Highest Education**				
Primary school	0 (0)	204 (90.3)	0 (0)	204 (61)
Secondary school	8 (11)	21(9.3)	0 (0)	29 (9)
Bachelor & Master	64 (90)	1(0.4)	38 (100)	103 (30)
**Workplace**				
District office	0 (0)	0 (0)	4 (10.5)	4 (1)
Sector office	0 (0)	0 (0)	34 (89.5)	34 (10)
Referral Hospital	55 (76)	0 (0)	0 (0)	55 (16)
Health center	17 (24)	0 (0)	0 (0)	17 (5)
Community	0 (0)	226 (100)	0 (0)	226 (67)
**Podoconiosis Case(s) seen**^**3**^	N = 70	N = 224	N/A	N = 294
0≥1	57 (81) 13 (19)	218 (97) 6 (3)	N/A	275 (94) 19 (6)

^1^FHCP: Formal Healthcare Professional (physicians, nurses, midwives)

^2^CHW: Community Health Worker

^3^Per month

N/A: Not applicable

### Knowledge about podoconiosis

The overall score for knowledge was 58.5%. More than half (60%) of participants knew that soil is a cause of podoconiosis, but only 44% identified soil as the only direct cause of podoconiosis. Some participants believed that podoconiosis was a hereditary or a randomly occurring disease (21% and 24%, respectively; Table 2). Most participants (61%) knew that walking barefoot was a risk factor for acquiring podoconiosis but only 17% knew that farmers are a high risk group. Slightly more than the half of respondents (51% compared to 37%) thought women were more likely to have podoconiosis than men. Additionally, 16% incorrectly believed that children belonged to the high risk group. Across all disciplines, 86% of participants correctly identified “irreversible foot or leg swelling at late stage” as a sign and the same percentage knew that podoconiosis was treatable. All other signs and symptoms were identified by less than 40% of respondents (Table 2).

**Table 2:** Study participant knowledge of podoconiosis in Musanze District, Rwan.

	Frequency N (%)			
Knowledge	FHCP^1^ (N = 72)	CHWs^2^ (N = 226)	Environmental officers (N = 38)	Overall (N = 336)
**Causes (“soil” is the correct answer)**				
Heredity	10 (14)	45 (20)	16(42)	71 (21)
Contact with affected patients	0 (100)	2 (1)	1 (3)	3 (1)
Mosquitoes	18 (25)	4 (2)	9 (24)	31 (9)
Soil	38 (53)	151 (67)	13 (34)	202 (60)
Spiritual cause/curse	0 (0)	7 (3)	1 (3)	8 (2)
Randomly occurring	12 (17)	68 (30)	0 (0)	80 (24)
Poverty	3 (4)	9 (4)	3 (8)	15 (4.5)
Overall score (%)				85.45
**High risk groups (“children” is the incorrect answer)**				
Adult men	18 (25)	94 (42)	13 (34)	125 (37)
Adult women	35 (49)	114 (50)	22 (58)	171 (51)
Children	5 (7)	43 (19)	5 (13)	53 (16)
Farmers	10 (14)	37 (16)	10 (26)	57 (17)
People who walk barefoot	43 (60)	142 (63)	20 (53)	205 (61)
People who don’t wash legs after soil contact	19 (26)	85 (38)	12 (32)	116 (34.5)
Overall score (%)				47.47
**Preventive measures (all correct except “avoiding marriage with patients and their families”)**				
Washing feet after contact with soil	45 (63)	167 (74)	28 (74)	240 (71)
Avoiding contact with patients	3 (4)	8 (3.5)	4 (10)	15 (4.5)
Avoiding walking barefoot in the cold	20 (28)	71 (31)	13 (34)	104 (31)
Wearing shoes	55 (76)	195 (86)	29 (76)	279 (83)
Avoiding marriage with patients and their families	1 (1)	15 (7)	4 (10)	20 (6)
Overall score (%)				82.61
**Treatable (“yes” is the correct answer)**				
Yes	56 (78)	195 (86)	31 (82)	282 (84)
No	13 (18)	26 (12)	4 (10)	43 (13)
I don’t know	3 (4)	5 (2)	3 (8)	11 (3)
**Signs and symptoms (all are correct except “Loss of sensation on foot”)**				
Reversible foot or leg swelling at early stage	18 (25)	68 (30)	10 (26)	96 (29)
Itching	22 (31)	77 (34)	15 (40)	114 (34)
Irreversible foot or leg swelling at late stage	58 (81)	197 (87)	30 (79)	285 (86)
Burning sensations	13 (18)	40 (18)	9 (24)	62 (19)
Lump growth/protrusions	10 (14)	36 (16)	6 (16)	52 (16)
Widening of foot	25 (35)	81 (36)	15 (40)	121 (36)
Loss of sensation on foot	24 (33)	58 (26)	11 (29)	93 (28)
Formation of skin folds	24 (33)	59 (26)	9 (24)	92 (28)
Knocking of big toes	14 (19)	63 (28)	8 (21)	85 (25.5)
Shallow skin folds	8 (11)	46 (20)	10 (26)	64 (19)
Plantar edema	5 (7)	26 (12)	5 (13)	36 (11)
Large second toes	6 (8)	42 (19)	6 (16)	54 (16)
Overall score (%)				31.51

^1^FHCP: Formal Healthcare Professional (physicians, nurses and midwives)

^2^CHW: Community Health Worker

Among formal health professionals, the average percentage of correctly answered questions ranged from 39% to 55% (mean = 55% SD = +/-12%). Among CHWs, the average percentage of correctly answered questions ranged from 36% to 94% (mean = 59% SD = +/-13.5%), while for environmental officers the average ranged from 30% to 94% (mean = 58% SD+/-12%) (Table 2).

Knowledge score was not associated with profession (p = 0.41), education level (p = 0.83), work experience (p = 0.311) or number of cases treated per month (p = 0.382) (Table 3).

**Table 3:** Chi-square analysis of podoconiosis knowledge among study participants surveyed in Musanze District, Rwanda.

		Low knowledge score (<80%)	High knowledge score (≥80%)	p-value
**Discipline**		**N (%)**		
	FHCP^1^	69 (22)	3 (11)	0.410
	CHW^2^	205 (67)	21 (78)	
	Environmental officers	35 (11)	3 (11)	
**Education level**				
	Primary	187 (60)	17 (64)	0.083
	Secondary	24 (8)	5 (18)	
	Bachelor& Master	98 (32)	5 (18)	
**Work experience**				
	<5 years	63 (20)	6 (22)	0.311
	5–10 years	123 (40)	7 (26)	
	>10 years	123 (40)	14 (52)	
**Cases treated per month**				
	0	251 (93)	24 (100)	0.382
	≥1	19 (7)	0 (0)	

^1^FHCP: Formal Healthcare Professional (physicians, nurses and midwives)

^2^CHW: Community Health Worker

### Attitudes toward podoconiosis

Overall, 86% of respondents expressed positive attitudes towards podoconiosis with the exception of four areas (Table 4). Namely, half of participants believed that podoconiosis patients had poor hygiene (41%) and that society provided enough help to people with podoconiosis (38%). Half of respondents (50%) believed that people who said they were podoconiosis patients are not brave and strong and more than two thirds (76%) believed that podoconiosis patients were a threat to their own health and their family’s health (Table 4).

**Table 4:** Study participant attitudes about podoconiosis in Musanze District, Rwanda.

	Frequency N (%)		
Attitude	Agree	Disagree	IDK^1^
**Health professionals N = 298**			
Health care providers are in danger of contracting podoconiosis	10 (3)	283 (95)	5 (2)
People will isolate me if they knew I treated podoconiosis patients	8 (3)	289 (97)	1 (0)
People will appreciate me if they knew I treated podoconiosis patients	293 (98)	5 (2)	0 (0)
People will isolate my family members if they knew I treated podoconiosis patient	4 (1)	294 (99)	0 (0)
**Health professionals and environmental officers N = 336**			
People get podoconiosis because they have sinned	3 (1)	331 (99)	0 (0)
Podoconiosis patients have poor hygiene	139 (41)	197 (59)	0 (0)
Podoconiosis patients deserves love and support	328 (98)	8 (2)	0 (0)
I buy food or items from a shopkeeper with podoconiosis	323 (96)	12(4)	1 (0)
I am comfortable if my food server has podoconiosis	306 (91)	25 (7)	5 (2)
I am at risk of acquiring podoconiosis if I am in contact with a podoconiosis patient	10 (3)	324 (96)	2 (1)
It is a person`s own fault if they develop podoconiosis	46 (14)	286 (85)	4 (1)
People with podoconiosis should be ashamed of themselves	30 (9)	306 (91)	0 (0)
People with podoconiosis can remain competitively productive members of society	246 (73)	89 (27)	1 (0)
Our society does not provide enough help to people with podoconiosis	146 (44)	129 (38)	61 (18)
People who say they are podoconiosis patients are brave and strong	167 (50)	168 (50)	1 (0)
People with podoconiosis are a threat to their own health and their family’s health	254 (76)	82 (24)	0 (0)
People with podoconiosis deserve sympathy	324 (97)	12 (4)	0 (0)
People with podoconiosis deserve treatment and care	332 (99)	4 (1)	0 (0)
The family of the person with podoconiosis should be blamed for passing on the disease	17 (5)	317 (94)	2 (1)
The family of the person with podoconiosis is cursed	4 (1)	332 (99)	0 (0)
The family of the person with podoconiosis should be isolated	1 (0)	335 (100)	0 (0)
People with podoconiosis should be legally separated from others to protect the public health	5 (2)	330 (98)	1 (0)

^1^IDK: I don’t know

Three factors were found to be statistically associated with attitude scores: discipline (p<0.001), education level (p<0.001), and work experience (p<0.001) (Table 5).

**Table 5:** Chi-square analysis of podoconiosis attitudes among study participants surveyed in Musanze District, Rwanda.

		Positive attitude	Negative attitude	p-value
**Discipline**				
	FHCP^1^	18 (29)	54 (20)	<0.001
	CHW^2^	22 (35)	204 (75)	
	Environmental officers	23 (36)	15 (5)	
**Education level**				
	Primary	21 (33)	183 (67)	<0.001
	Secondary	2 (3)	27 (10)	
	Tertiary	40 (64)	63 (23)	
**Work experience**				
	<5 years	24 (38)	45 (16)	<0.001
	5–10 years	22 (35)	108 (40)	
	>10 years	17 (27)	120 (44)	
**Cases seen per month**				
	0	37 (93)	238 (94)	0.493
	≥1	3 (7)	16 (6)	
**Knowledge score**				
	≥80%	8 (13)	19 (7)	0.131
	<80%	55 (87)	254 (93)	

^1^FHCP: Formal Healthcare Professional (physicians, nurses, midwives)

^2^CHW: Community Health Worker

### Podoconiosis management practices

The overall score for practices ranged from 25% to 100% (mean+72%, SD+/-14%). Regarding the management of acute cases, few health professionals (5%) knew that they should prescribe antibiotics but most knew that they could refer such patients to higher health facilities. Approximately one-fifth (14%) incorrectly thought that podoconiosis required laboratory investigations. One-third of health providers (32%) knew that ointment and soap are topical treatments recommended in the management of podoconiosis (Table 6).

**Table 6:** Podoconiosis management practices reported by formal healthcare professionals and community health workers in Musanze District, Rwanda.

	Frequency N (%)			
Practices	FHCP^1^ (N = 72)	CHWs^2^ (N = 226)	Overall N = 298	Answer
**Management of acute attack**				
Prescribed antibiotics	14 (19)	1 (0)	15(5)	correct
Surgical treatment	2 (3)	0 (0)	2(1)	incorrect
Prescribed DEC^3^	6 (8)	0 (0)	6(2)	incorrect
Lab investigations	38 (53)	3 (1)	41(14)	incorrect
Referral to other health facility	50 (69)	215 (95)	265(89)	correct
**Management of chronic condition**				
Topical ointments/soaps	22 (31)	74 (33)	96(32)	correct
Surgical treatment	4 (6)	1 (0)	5(2)	incorrect
Referral to other health facility	57 (79)	211 (93)	268(90)	correct

^1^FHCP: Formal Healthcare Professional (physicians, nurses, midwives)

^2^CHW: Community Health Worker

^3^DEC: Diethylcarbamazine

Respondents identified medicine and supply shortages as the primary challenge in managing podoconiosis (84%), followed by treatment refusal (15%), unpleasant odor (2%), discomfort at workplace (1%), and high workload (1%).

## Discussion

To our knowledge, this study was the first to assess the KAP of health professionals and environmental officers toward podoconiosis in Rwanda. Despite the high prevalence of podoconiosis in Musanze district, knowledge levels were low across all respondents, and only 6% of health professionals had treated patients with this disease. Only 44% of respondents correctly identified soil as the only direct cause of podoconiosis, 21% knew it was a hereditary condition, and most respondents were unaware of signs and symptoms other than swelling of the legs. Understanding that it is susceptibility to podoconiosis that is inherited, rather than the condition itself, is conceptually difficult, and, like health professionals in Ethiopia [11], our respondents might not understand the role of heritability clearly. This gap in health professional knowledge is concerning because early diagnosis is a key factor contributing to favorable prognosis [18].

Our results also suggest that the countrywide NTD training provided to CHWs in 2018 did not sufficiently improve knowledge of podoconiosis. Furthermore, education level was not significantly associated with knowledge score, indicating that physicians and nurses were in equal need of training on this topic. Studies conducted in Cameroon and Ethiopia demonstrated that CHWs and selected podoconiosis patients (i.e. Community Podoconiosis Agents (CPAs)) could successfully treat fellow podoconiosis patients given appropriate training and supervision [14,19]. Other studies have also shown that CHWs have the ability to participate in patient care but that CPAs are challenged when podoconiosis patients need care for other diseases [14,20]. Given that CHWs live in close proximity to patients and are familiar with the health system, well-trained CPAs supervised by CHWs could potentially be a cost efficient means to manage podoconiosis.

Knowledge of risk factors and preventive measures was inconsistent. While 17% of respondents recognized farmers to be the most affected group, most correctly identified protective measures including wearing shoes (83%) and washing feet after contact with soil (71.4%). Many respondents believed that women were more likely to contract podoconiosis than men, although there is no biological reason for differences in prevalence [4]. However, in Ethiopia, Uganda and Kenya, disparities in income and access to personal protective equipment are apparent between men and women, especially in rural, agricultural areas, providing an explanation for why women were more affected [5,21,22].

On one hand, most respondents had positive attitudes toward podoconiosis patients and the majority of health professionals were not afraid of treating podoconiosis or worried about receiving criticism for taking care of affected patients. On the other hand, the majority of respondents considered podoconiosis patients to be a threat to their own health and to that of their family. These contradictory beliefs imply that health providers ignored both the value of treatment and the nature of the disease. It is important to further investigate the reasons behind this paradoxical knowledge in order to combat this stigma. Our results were supported by a study from southern Ethiopia demonstrating that almost all health professionals held at least one stigmatizing attitude or misconception about podoconiosis [11].

In our study, the three factors associated with positive attitude with a percentage of 80% and above about attitude questions, were occupation–more CHWs had positive attitudes; education level–more who had completed primary school had positive attitudes; and work experience–those with longer work experience had more positive attitudes. However, a previous study in Ethiopia showed that positive attitudes toward podoconiosis were associated with good knowledge [11]. CHWs, most of whom had primary education, made up the majority of our respondents. It is possible that they held a more positive attitude due to their close proximity to affected patients. Since few had regularly treated patients with the disease, respondents with more work experience probably had more cumulative contact and had thus developed better attitudes. Previous studies in Ethiopia have also shown that using vigorous sensitization campaigns including via radio, television, social media as well as using public figures as podoconiosis advocates did increase the knowledge and support of the general population toward podoconiosis [23]. By demystifying false beliefs and illusions, more positive attitudes toward podoconiosis are gradually developed in endemic communities.

Overall scores for managing podoconiosis patients were low. This is similar to Ethiopia, where the majority of health professionals did not have sufficient confidence in their skills and knowledge to provide standard treatment to patients with podoconiosis [11]. The two areas where practices scored lowest were related to the provision of medical supplies—providing antibiotics in acute cases and moisturizing ointments to prevent skin cracking in chronic cases. Additionally, 84% of health professionals reported lack of medications and supplies as the number one challenge in treating podoconiosis. Systems to ensure the needed supplies and medications for treatment should be established and indications for treatment should be included in the national essential medicines list of every endemic country. This would increase quality of life for affected people and reduce economic losses associated with this condition [11].

Fifteen percent of health professionals reported that patients refusing treatment was a key challenge they faced in the management of podoconiosis, and only 6% reported seeing at least one case per month. There is no published literature on treatment refusal in Rwanda; however, long distances to health facilities, stigma, and high expectations regarding treatment, supportive aid, discrimination and self-stigma were identified as factors affecting care seeking behavior in Ethiopia [1,24].

Our study obtained diverse perspectives by uniquely and intentionally including environmental officers to ensure consistent messaging in prevention of this disease. Podoconiosis is a neglected and complex health problem, involving medical, environmental and behavioral components and requires multidisciplinary collaboration for prevention and management [25]. Affected patients are mostly very poor farmers who live far from health centers, usually unable to work and very stigmatized by their communities, which sometimes lead to mental health problems [26]. Altogether, these factors instill self-stigma and lack of confidence among patients which eventually discourage them from seeking healthcare [1]. Well informed environmental officers could play a key role in identifying factors such as pathogenic soil minerals embedded in volcanic rocks, temperature and precipitations levels that favor the occurrence of podoconiosis [27]. Consequently, the development of comprehensive podoconiosis prevention strategies targeting environmental factors found in podoconiosis endemic zones could be achieved [27]. Furthermore, the proximity between environmental officers and subsistence farmers put them in good position to both encourage safe farming practices such as use of protective footwear and spread preventive messages about podoconiosis in a more direct and effective manner than health professionals.

The results must be seen in light of some limitations. In addition to its cross sectional design, the sample sizes of physicians, nurses, and environmental officers were small compared to CHWs and only one district (Musanze) among 30 in Rwanda was selected for this study. The study design also could not confirm the accuracy of self-reported survey data. Despite those limitations, our findings have demonstrated the importance of future qualitative studies, which will elucidate the contextual dynamics associated with the knowledge gap toward podoconiosis which was found among health professionals and environmental officers in Rwanda.

We identified barriers to optimizing healthcare for podoconiosis patients that will inform policy makers including the Rwandan Ministries of Health, Agricultural and Environment as they develop evidence-based interventions for the control of podoconiosis. As examples, strengthening medical curricula will allow the adequate training of health professionals who are the main building blocks of an effective health system [28,29] and provision of in service capacity building for health professionals and environmental officers will help to ensure productive and complementary collaboration in the control of podoconiosis. Policymakers also need to bring equity at the center of podoconiosis control strategies by making the supplies necessary—such as soap, ointments and bandages—for podoconiosis management available at the community level, to ensure continuity and sustainability in the treatment of podoconiosis patients who are most of the time unable to afford those supplies by themselves as it was demonstrated in Ethiopia [14,23].

We finally recommend future studies to assess patients’ experiences and barriers to their health seeking behavior which together with our findings will allow the NTD program to implement proper policy toward podoconiosis in Rwanda and enrich the body of knowledge for this stigmatized and debilitating condition.
